# Studying the Comorbidity of Impaired eGFR With Diabetes, Hypertension, and Cardiovascular Diseases in the Tabari Cohort Population

**DOI:** 10.1002/puh2.70385

**Published:** 2026-09-23

**Authors:** Amirhossein Hessami, Farhad Gholami, Motahareh Kheradmand, Reza Alizade‐Navaei, Mahmood Moosazadeh

**Affiliations:** ^1^ Non‐Communicable Disease Institute Mazandaran University of Medical Sciences Sari Iran; ^2^ Department of Internal Medicine, Faculty of Medicine Mazandaran University of Medical Sciences Sari Iran; ^3^ Health Science Research Center Mazandaran University of Medical Sciences Sari Iran; ^4^ Gastrointestinal Cancer Research Center, Non‐Communicable Disease Institute Mazandaran University of Medical Sciences Sari Iran

**Keywords:** cardiovascular diseases, comorbidity, diabetes mellitus, hypertension, reduced estimated glomerular filtration rate (eGFR), Tabari cohort

## Abstract

**Background:**

This study examines the prevalence and interrelationships of reduced estimated glomerular filtration rate (eGFR) with diabetes mellitus (DM), hypertension (HTN), and cardiovascular diseases (CVDs) within the Tabari cohort population.

**Methods:**

A cross‐sectional analysis was performed using data from the Tabari cohort, part of the large Prospective Epidemiological Research Studies in Iran (PERSIAN) cohort study. Participants aged 35–70, excluding those with end‐stage renal disease or cancer, were included. Reduced eGFR was defined on the basis of a single baseline measurement of eGFR, categorized into G1–G4 stages. The presence of DM, HTN, and CVD was recorded along with demographic and clinical parameters. Statistical analyses included chi‐square tests, *t*‐tests, and multiple logistic regressions.

**Results:**

Of 9939 participants, 25.5% had reduced eGFR, with prevalence rates of 4.8%, 69.7%, 25.4%, and 0.1% for G1, G2, G3, and G4, respectively. Reduced eGFR was more common with increasing age, in females, and in single/divorced/widowed individuals (*p* < 0.001). Higher prevalence was also observed among those with lower educational and socioeconomic status, urban residence, retirement or housewife occupational status, and elevated body mass index (BMI), waist circumference, triglycerides, and cholesterol (*p* < 0.001). HTN, DM, and CVD were more frequent in individuals with reduced eGFR, with increasing prevalence across declining eGFR categories. After adjustment, HTN remained significantly associated with reduced eGFR (adjusted OR = 1.36, 95% CI: 1.21–1.52), whereas associations for DM and CVD were attenuated.

**Conclusion:**

A high comorbid prevalence of HTN, DM, and CVD was observed among individuals with reduced eGFR in the Tabari cohort, with HTN emerging as the strongest independent predictor. These findings highlight the importance of early detection and integrated management strategies to prevent further decline in kidney function and associated complications.

## Graphical abstract

Among 9939 adults from the Tabari cohort, 25.5% had impaired eGFR. Although diabetes, hypertension, and cardiovascular disease were more common with declining kidney function, only hypertension remained independently associated with impaired eGFR.

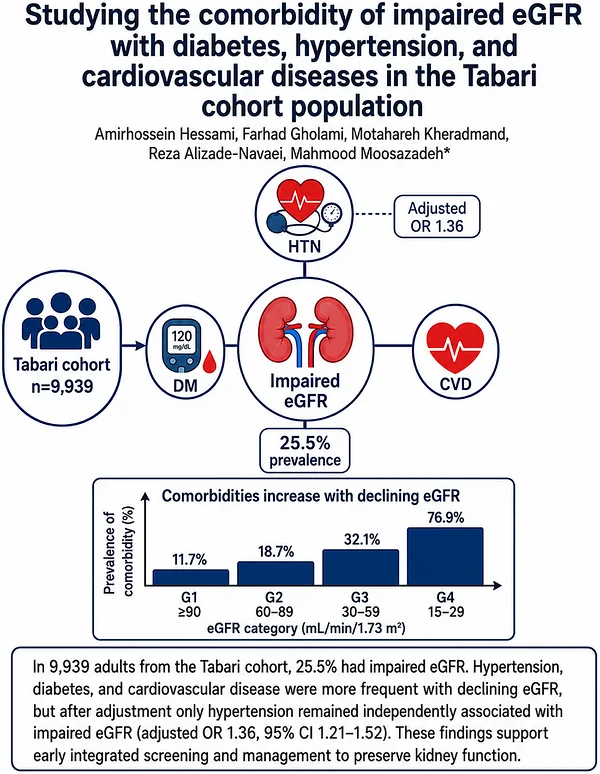

## 1 Introduction

Reduced kidney function, often reflected by a decline in estimated glomerular filtration rate (eGFR), represents a significant public health concern worldwide. Although chronic kidney disease (CKD) is clinically defined by persistent kidney abnormalities for at least 3 months, reduced eGFR at a single time point is used low‐income CKD programs as an important indicator of impaired renal filtration, cardiometabolic risk, and future kidney‐related outcomes [1, 2]. CKD stands as one of the major global causes of mortality and morbidity, affecting more than 10% prevalence and responsible for 1.2 million deaths among general population [3, 4]. World Health Organization estimates the CKD mortality will increase to 14 deaths per 100,000 in 2030 [5]. In a previous meta‐analysis study in 2016, the prevalence of CKD was estimated to be 11.6% in Iran [6] with more than 10,000 deaths in Iran in 2017 according to Global Burden of Disease study [4].

Impaired GFR is recognized as an independent risk factor for cardiovascular diseases (CVDs), with all stages of the condition associated with heightened risks of cardiovascular complications, premature mortality, and reduced quality of life [6]. The presence of diabetes and hypertension (HTN) further accentuates the cardiovascular risk profile in CKD patients, necessitating vigilant management strategies to mitigate adverse outcomes [7]. Previous cross‐sectional studies have indicated that over a quarter of patients with diabetes and more than a fifth of those with HTN suffer from CKD [8]. Research conducted among a large and diverse adult population has demonstrated a significant association between a decline in GFR and increased risks of mortality, cardiovascular events, and hospitalization, independent of established risk factors, along with the presence of proteinuria [9].

HTN is also a major contributor to impaired GFR through its hemodynamic effects on the renal vasculature, leading to glomerular injury and subsequent decline in renal function [10]. Conversely, impaired GFR exacerbates HTN by disrupting renal sodium handling and the renin–angiotensin–aldosterone system, thereby perpetuating a vicious cycle of kidney damage and elevated blood pressure [11]. The coexistence of HTN and CKD substantially amplifies the risk of cardiovascular events, underscoring the complex interplay between these conditions [12].

Given the complex interactions between impaired GFR, diabetes, HTN, and cardiovascular disorders, comprehensive population‐based studies are warranted to explain the epidemiological patterns and prognostic implications of these comorbidities. The Tabari cohort, as part of the large Prospective Epidemiological Research Studies in Iran (PERSIAN), provides a unique opportunity to assess kidney function alongside a wide range of demographic, clinical, and lifestyle variables. However, limited evidence exists regarding the prevalence of reduced eGFR and its comorbid patterns, especially in Northern Iran, where cardiometabolic risk factors are highly prevalent. Therefore, the present study aims to investigate the prevalence of reduced eGFR and its associations with diabetes, HTN, and CVDs in the Tabari cohort population.

## 2 Methods and Materials

### 2.1 Study Design, Setting, and Population

This cross‐sectional study was done using data collected during enrollment of Tabari cohort study [13]. The enrollment phase of the study was carried out between June 1, 2015, and November 7, 2017. The Tabari cohort is a subset of the larger nationwide cohort study known as the PERSIAN cohort. Details regarding the rationale, objectives, and design of the PERSIAN cohort can be found in previously published papers [14, 15].

Inclusion criteria encompassed individuals aged 35–70 living within the study area. Exclusion criteria comprised individuals declining participation. Additionally, individuals unable to communicate effectively, including those who were deaf, blind, mute, or paralyzed but could not access the cohort center, diagnosed with cancer and ESRD (eGFR < 15 mL/min/1.73 m^2^, G5), were excluded.

### 2.2 Data Collection and Laboratory Measurements

Blood and urine samples were collected from all participants during the baseline assessment of the Tabari cohort study. Kidney function was assessed using eGFR, calculated from serum creatinine measurements using the 2021 CKD‐EPI creatinine equation. According to Kidney Disease: Improving Global Outcomes (KDIGO) recommendations, eGFR categories were classified as G1 (≥90 mL/min/1.73 m^2^), G2 (60–89 mL/min/1.73 m^2^), G3 (30–59 mL/min/1.73 m^2^), and G4 (15–29 mL/min/1.73 m^2^) [16]. Impaired eGFR was defined as eGFR < 60 mL/min/1.73 m^2^. As only baseline measurements were available, impaired eGFR was considered an indicator of reduced kidney function rather than confirmed CKD, which requires persistence for at least 3 months [17]. Additionally, the presence of kidney stones and kidney failure was determined on the basis of self‐reported information provided by the participants.

The variables recorded included age, gender, area of residence, level of education, occupation, presence of diabetes, HTN, and CVDs (including myocardial infarction, heart failure, and arrhythmia). Furthermore, measurements of fasting blood sugar levels (FBS), systolic and diastolic blood pressure (DBP), lipid profile, body mass index (BMI), waist and hip circumferences, and ratios were included in the analysis. eGFR was categorized into five stages, including ≥90 (elevated or normal, G1), 60–89 (mild reduction, G2), 30–59 (moderate reduction, G3), and 15–29 (severe reduction, G4) [18]. HTN was defined by a systolic blood pressure (SBP) of 140 mmHg or higher, a DBP of 90 mmHg or higher, a history of high blood pressure, or current treatment with antihypertensive medications [19]. Diabetes mellitus (DM) was defined as FBS ≥ 126 mg/dL following 12 h of fasting or a history of diagnosis or use of glucose‐lowering medication [20]. CVD was defined according to the Tabari cohort protocol on the basis of documented physician diagnosis and medical history. Participants were considered to have CVD if they reported a history of coronary heart disease, including stable angina pectoris, unstable angina, previous myocardial infarction, heart failure, percutaneous coronary intervention, or coronary artery bypass graft surgery. Self‐reported cardiovascular conditions were verified through review of available medical records by physicians.

### 2.3 Statistical Analysis

The data were initially provided in Excel format. Afterward, the variables were imported into SPSS software version 26. Qualitative variables were summarized with number and percentage, and quantitative variables were described using mean and standard deviations. Demographic and epidemiological variables were compared between individuals with normal and impaired eGFR using the chi‐square test. A *t‐*test was also used comparing the clinical and laboratorial measurements. Moreover, multiple logistic regression was used to adjust for confounding variables and explore the association between impaired eGFR and comorbid conditions.

## 3 Results

The study comprised a total of 9939 participants, among whom 5896 (59.3%) were females. A total of 6896 (69.4%) resided in urban areas, and 3372 (33.9%) were aged 40–49 years. A number of 9133 individuals were classified married, and 2822 (28.4%) reported having received 9–12 years of education. Notably, 2173 were diagnosed (21.9%) with HTN, followed by 884 (8.9%) with CVD, 1686 (17.0%) with DM, and 185 (1.9%) presenting all three comorbid conditions simultaneously (Table 1).

**TABLE 1:** Characteristics of participants included into the study.

Characteristics	Total population (*N* = 9939), *n* (%)	Impaired eGFR (*N* = 2537), *n* (%)	Normal eGFR (*N* = 7402), *n* (%)	*p* value
Age				
35–39	1599	183 (11.4)	1416 (88.6)	
40–49	3372	647 (19.2)	2725 (80.8)	<0.001
50–59	3108	939 (30.2)	2169 (69.8)	
60–70	1860	768 (41.3)	1092 (58.7)	
Gender				
Male	4043	873 (21.6)	3170 (78.4)	<0.001
Female	5896	1664 (28.2)	4232 (71.8)	
Marital status				
Single/Divorced/Widowed	806	296 (33.4)	537 (66.6)	<0.001
Married	9133	2268 (24.8)	6865 (75.2)	
Education				
No schooling	1430	471 (32.9)	959 (67.1)	
1–5 years	2250	513 (22.8)	1737 (77.2)	
6–8 years	1094	231 (21.1)	863 (78.9)	<0.001
9–12	2822	701 (24.8)	2121 (75.2)	
University/College	2343	621 (26.5)	1722 (73.5)	
Socioeconomical status				
I	1937	454 (23.4)	1483 (76.6)	
II	1969	452 (23.0)	1517 (77.0)	
III	2005	499 (24.9)	1506 (75.1)	<0.001
IV	2016	515 (25.5)	1501 (74.5)	
V	2012	617 (30.7)	1395 (69.3)	
Residence				
Urban	6896	1834 (26.6)	5062 (73.4)	<0.001
Rural	3043	703 (23.1)	2340 (76.9)	
Employment status				
Employed	4294	871 (20.3)	3423 (79.7)	
Housewife	4566	1271 (27.8)	3295 (72.2)	<0.001
Jobless	101	26 (25.7)	75 (74.3)	
Retired or broken‐down	978	369 (37.7)	609 (62.3)	
Smoking				
Yes	905	195 (21.5)	710 (78.5)	<0.001
No	9034	2342 (25.9)	6692 (74.1)	
BMI (kg/m^2^)				
<25	2379	421 (17.7)	1958 (82.3)	
25–29.9	4221	1133 (26.8)	3088 (73.2)	<0.001
≥30	3339	983 (29.4)	2356 (70.6)	
WC (cm)				
<102 in male or <88 female	5077	1102 (21.7)	3975 (78.3)	
≥102 in male and ≥88 for female	4862	1435 (29.5)	3427 (70.5)	<0.001
WHR				
≤0.9 in male or ≤0.85 in female	3145	647 (20.5)	2507 (79.5)	
>0.9 in male or >0.85 in female	6785	1890 (27.9)	4895 (72.1)	<0.001
WHtR				
<0.5	1345	219 (16.3)	1126 (83.7)	<0.001
≥0.5	8594	2318 (27.0)	6276 (84.8)	
TG (mg/dL)				
<150	5200	1126 (21.7)	4074 (78.3)	<0.001
≥150	4793	1411 (29.8)	3328 (70.2)	
TC (mg/dL)				
<200	5381	1029 (19.1)	4325 (80.9)	<0.001
≥200	4558	1508 (33.1)	3050 (66.9)	
HDL (mg/dL)				
≥40 in male or ≥50 in female	6584	1685 (25.6)	4899 (74.4)	
<40 in male or <50 in female	3355	852 (25.4)	2503 (74.6)	0.846
Comorbidities				
HTN	2173 (21.9)	819 (32.3)	1354 (18.3)	
CVD	884 (8.9)	329 (13.0)	555 (7.5)	<0.001
DM	1686 (17.0)	589 (23.2)	1097 (14.8)	
DM + HTN + CVD	185 (1.9)	84 (0.8)	101 (1.4)	

*Note: p* values represent comparisons between participants with impaired eGFR and normal eGFR. Continuous variables are reported as mean ± standard deviation, and categorical variables as number (percentage).

Abbreviations: BMI, body mass index; CVD, cardiovascular disease; DM, diabetes mellitus; eGFR, estimated glomerular filtration rate; HDL, high‐density lipoprotein; HTN, hypertension; TC, total cholesterol; TG, triglycerides; WC, waist circumference; WHR, waist‐to‐hip ratio; WHtR, waist‐to‐height ratio.

The overall estimated prevalence of impaired eGFR was found to be 25.5%. The prevalence for stages G1, G2, G3, and G4 has prevalence rates of 4.8%, 69.7%, 25.4%, and 0.1%, respectively. The prevalence of impaired eGFR increased with age and was higher in females (28.2%) than males (21.6%), as well as more common in single/divorced/widowed individuals (33.4%) compared to married subjects (24.8%) (*p* < 0.001). Lower education and socioeconomic status, urban residence, and being retired or a housewife were associated with higher rates of impaired eGFR. Non‐smokers, higher BMI, WC, WHR, WHtR, elevated triglycerides, and total cholesterol were also associated with increased impaired eGFR prevalence (all *p* < 0.001). However, HDL cholesterol levels showed no significant difference (*p* = 0.846) (Table 1).

The prevalence of comorbid conditions, including HTN, DM, and CVD, was significantly higher in patients with impaired eGFR with 21.9%, 17.0%, and 8.9%, respectively.

The analysis of clinical traits showed FBS was significantly higher in patients with impaired eGFR compared with normal kidney function subjects (114.55 ± 35.30 vs. 108.37 ± 31.77, *p* < 0.001). There was also significantly higher SBP and DBP among patients with impaired eGFR (Table 2).

**TABLE 2:** Clinical and laboratorial factors of individuals included into the study.

Clinical and laboratorial parameters	Impaired eGFR, mean ± SD	Normal eGFR, mean ± SD	*p* value
FBS (mg/dL)	114.55 ± 35.30	108.37 ± 31.77	<0.001
SBP (mmHg)	147 ± 12	132 ± 11	<0.001
DBP (mmHg)	74 ± 8	73 ± 7	<0.001

Abbreviations: DBP, diastolic blood pressure; eGFR, estimated glomerular filtration rate; FBS, fasting blood sugar; SBP, systolic blood pressure.

HTN prevalence increased significantly from 11.7% in G1 to 76.9% in G4. Similarly, DM showed a rising trend from 12.3% in G1 to 61.5% in G4. CVD prevalence also significantly increased from 5.9% in G1 to 12.9% in G3, with a drop to 0.5% in G4. The combined prevalence of DM, HTN, and CVD escalates significantly from 0.4% in G1 to 23.1% in G4. These findings indicate a significant association between declining eGFR and the increased prevalence of these comorbid conditions (Table 3).

**TABLE 3:** Prevalence of comorbid conditions based on GFR categories.

	G1 (*N* = 478), *n* (%)	G2 (*N* = 6924), *n* (%)	G3 (*N* = 2521), *n* (%)	G4 (*N* = 13), *n* (%)	*p* value
HTN	56 (11.7)	1298 (18.7)	809 (32.1)	10 (76.9)	<0.05
DM	59 (12.3)	1038 (15.0)	581 (23.0)	8 (61.5)	<0.05
CVD	28 (5.9)	527 (7.6)	325 (12.9)	4 (0.5)	<0.05
DM + HTN + CVD	2 (0.4)	99 (1.4)	81 (3.2)	3 (23.1)	<0.05

*Note:* GFR categories: G1: ≥90 (elevated or normal), G2: 60–89 (mild reduction), G3: 30–59 (moderate reduction), and G4: 15–29 (severe reduction).

Abbreviations: CVD, cardiovascular disease; DM, diabetes mellitus; HTN, hypertension.

In the unadjusted analysis, DM was associated with significantly increased odds of impaired eGFR (OR = 1.73, 95% CI: 1.55–1.94, *p* < 0.05). However, after adjusting for the covariates, the association was attenuated and was no longer statistically significant (adjusted OR = 1.13, 95% CI: 0.99–1.27, *p* = 0.052). HTN showed a significant association with impaired eGFR in both unadjusted (OR = 2.12, 95% CI: 1.92–2.35, *p* < 0.05) and adjusted models (Adjusted OR = 1.36, 95% CI: 1.21–1.52, *p* < 0.05), indicating that HTN remains a significant predictor of impaired eGFR even after controlling for other factors. CVD was also significantly associated with impaired eGFR in the unadjusted model (OR = 1.83, 95% CI: 1.59–2.12, *p* < 0.05). This association became nonsignificant in the adjusted model (adjusted OR = 1.13, 95% CI: 0.97–1.33, *p* = 0.111) (Table 4).

**TABLE 4:** Univariate and multivariate logistic regression analysis of comorbid conditions as predictors of impaired estimated glomerular filtration rate (eGFR).

Comorbid condition	Unadjusted OR (95% CI)	*p* value	Adjusted OR (95% CI)^a^	*p* value
DM	1.73 (1.55–1.94)	<0.05	1.07 (0.95–1.22)	0.243
HTN	2.12 (1.92–2.35)	<0.05	1.35 (1.20–1.51)	<0.05
CVD	1.83 (1.59–2.12)	<0.05	1.06 (0.90–1.24)	0.474
DM + HTN + CVD	2.47 (1.84–3.31)	<0.05	1.28 (0.93–1.75)	0.120

Abbreviations: CVD, cardiovascular disease; DM, diabetes mellitus; HTN, hypertension.

^a^ Adjusted for residence, smoking status, HDL level, WC, WHR, WHtR, BMI, education, marital status, age, TG, TC, employment status, and gender.

For individuals with all three conditions (DM, HTN, and CVD), the unadjusted analysis indicated significant odds of impaired eGFR (OR = 2.47, 95% CI: 1.84–3.31, *p* < 0.05). After adjusting for the covariates, the odds ratio was reduced and did not reach statistical significance (adjusted OR = 1.28, 95% CI: 0.93–1.75, *p* = 0.120).

## 4 Discussion

Our study investigated the comorbidities of impaired eGFR with DM, HTN, and CVDs within the Tabari cohort population. The results of study showed that the prevalence of three mentioned comorbid conditions was significantly higher in patients with impaired eGFR. Studying the clinical and laboratorial parameters showed significantly higher FBS and SBP, DBP.

The overall estimated prevalence of impaired eGFR in Tabari cohort study was found to be 25.5%, with specific prevalence rates for stages G4, G1, G3, and G2 at 0.1%, 4.8%, 25.4%, and 69.7%, respectively. Previous meta‐analysis study in Asia showed 7.0% to 34.3% variation in CKD prevalence with pooled prevalence of 10.6% [21], placing our cohort in high rates of reduced eGFR. This variation in prevalence throughout Asia is due to substantial differences in etiologies, sociodemographic, and lifestyle factors [21].

Our data underscore the demographic disparities in prevalence of impaired kidney function. Patients diagnosed with reduced eGFR tended to be older, with a substantial portion falling within the 50–59 and 60–70‐year age groups. This age distribution aligns with existing literature that associates advancing age with increased risk of CKD due to the natural decline in renal function and higher likelihood of comorbid conditions [22]. The study also highlights a higher prevalence of impaired eGFR among females, which is consistent with some epidemiological studies that suggest gender differences in CKD prevalence. These differences may be attributed to various factors, including hormonal influences, differences in muscle mass, and healthcare‐seeking behaviors [23].

Socioeconomic factors were found to play a significant role, with lower socioeconomic status and urban residence being more prevalent among patients with impaired kidney function. Lower socioeconomic status is often associated with reduced access to healthcare, poor nutrition, and increased exposure to reduced kidney function risk factors such as HTN and diabetes [24]. Urban residence, on the other hand, may reflect environmental and lifestyle factors that contribute to the higher prevalence of this condition in these areas [25].

The study revealed that HTN was the most prevalent comorbid condition, especially in stage G4, with a prevalence of 76.9%. DM was most prevalent in stage G2 at 61.6%, whereas CVD showed a notable presence in stage G3 at 12.9%. Additionally, the co‐occurrence of all three conditions (HTN, DM, and CVD) was highest in stage G4 at 23.1%. These findings align with previous research indicating that patients with impaired GFR often present with multiple comorbidities, which complicate disease management and outcomes [26].

The bidirectional relationship between diabetes and reduced kidney function is well‐documented, with diabetic nephropathy being a leading cause of ESRD globally [27]. In the current study, unadjusted analysis, DM was associated with significantly increased odds of impaired GFR. However, this association was attenuated after adjusting for covariates, resulting in a nonsignificant adjusted odds ratio. This attenuation suggests that other factors such as age, socioeconomic status, and additional health conditions may play a role in the progression of reduced kidney function in diabetic patients [24, 28].

HTN demonstrated a significant association with impaired GFR in both unadjusted and adjusted models. Studies in Asian countries also showed 64.5% and 38.4% of CKD patients were diagnosed with HTN in India [29] and Malaysia [30], respectively. The Chronic Renal Insufficiency Cohort (CRIC) study also reported high rates of HTN in CKD patients, with significant implications for cardiovascular health. The interplay between CKD and HTN exacerbates cardiovascular risk, necessitating integrated management approaches [12, 31]. This consistent significance underscores HTN as a persistent and significant predictor of CKD [32]. The pathophysiological relationship between HTN and reduced kidney function is bidirectional, where HTN can cause kidney damage and CKD can exacerbate HTN [33]. Our findings are consistent with global research on CKD comorbidities. For instance, a study by Kovesdy highlights that HTN is a significant risk factor for CKD progression, mirroring our observations of its high prevalence among CKD patients [3].

CVD was significantly associated with impaired eGFR in the unadjusted model. However, similar to diabetes, this association became nonsignificant after adjustment for covariates. This result indicates that although CVD is prevalent among patients with impaired kidney function, its direct impact risk is likely influenced by overlapping risk factors and comorbid conditions [34]. These overlapping factors include traditional cardiovascular risk factors such as HTN and diabetes, as well as CKD‐specific factors such as mineral and bone disorders, which further complicate the cardiovascular risk profile in these patients [35].

For individuals with all three comorbid conditions (DM, HTN, and CVD), the unadjusted analysis indicated significantly increased odds of impaired eGFR. The interaction between these conditions and reduced kidney function suggests a complex interplay where each condition exacerbates the risk and progression of CKD, and managing these conditions collectively is crucial for patient outcomes [36]. Moreover, non‐traditional risk factors, such as inflammation and oxidative stress, also play significant roles in this interplay [37], necessitating a comprehensive approach to risk management in CKD patients. The observed coexistence of HTN, diabetes, and CVD with impaired GFR highlights the potential importance of comprehensive and integrated management approaches. These strategies may include early detection, control of blood pressure and glucose levels, and consideration of medications such as sodium‐glucose cotransporter 2 (SGLT2) inhibitors and glucagon‐like peptide‐1 receptor (GLP1) agonists, which have shown promise in reducing both cardiovascular and renal events in diabetic patients [38].

Several limitations should be considered when interpreting the findings of this study. First, because this analysis was based on a cross‐sectional design, temporal relationships and causal inferences between impaired eGFR and cardiometabolic comorbidities, including HTN, DM, and CVDs, cannot be established. Second, kidney function was assessed using a single baseline measurement of serum creatinine and eGFR; therefore, the presence of CKD could not be confirmed according to KDIGO criteria, which require evidence of kidney abnormalities persisting for at least 3 months. In addition, complete CKD classification based on the KDIGO Cause–GFR–Albuminuria (CGA) framework was not possible because information regarding urinary albumin‐to‐creatinine ratio (ACR) or other measures of albuminuria was unavailable in the current Tabari cohort dataset. Consequently, this study evaluated impaired eGFR as an indicator of reduced kidney function rather than providing a definitive estimate of CKD prevalence.

The high prevalence of impaired eGFR and its comorbidities in our cohort highlights the importance of early detection and integrated management approaches to mitigate CKD progression and associated complications. Future research should focus on developing targeted interventions to manage CKD and its comorbid conditions more effectively.

## 5 Conclusion

In conclusion, the study highlights the high prevalence of HTN, diabetes, and CVD among patients with impaired eGFR. HTN remains a significant predictor of GFR impairment even after adjusting for other factors, underscoring the need for effective blood pressure management in these patients. Although diabetes and CVD also show strong associations with reduced GFR, their impact appears to be moderated by other variables. This underscores the importance of a comprehensive approach in managing kidney function, taking into account the multifaceted interactions of these comorbid conditions.

## Author Contributions

**Amirhossein Hessami** contributed to the conceptualization of the study, data curation, statistical analysis, interpretation of findings, and drafting of the manuscript. **Farhad Gholami** contributed to clinical guidance, validation of clinical variables, and critical revision of the manuscript. **Motahareh Kheradmand** participated in the study design and coordination of data acquisition from the Tabari cohort database and contributed to reviewing and editing the manuscript. **Reza Alizade‐Navaei** contributed to the methodological framework, statistical supervision, and interpretation of analytical results and reviewed the manuscript for scientific accuracy. **Mahmood Moosazadeh** supervised the entire project, including conceptual design, methodology, overall data quality assurance, and interpretation of results. All authors have reviewed and approved the final manuscript.

## Funding

This project has been financially supported by Mazandaran University of Medical Sciences (Code: 17217).

## Ethics Statement

The study obtained approval from the Research Ethics Committee of Mazandaran University of Medical Sciences (IR.MAZUMS.IMAMHOSPITAL.REC.1401.068). Anonymity was strictly maintained throughout the research process. Authors had no access to information identifying individuals during and after the study. All methods were carried out in accordance with relevant guidelines and regulations.

## Consent

Informed consent was obtained from all participants, who were assured of the voluntary nature of their participation and their right to withdraw from the study at any time.

## Conflicts of Interest

The authors declare no conflicts of interest.

## Data Availability

The datasets generated and analyzed during the current study are not publicly available due to privacy and regulatory restrictions but are available from the corresponding author on reasonable request.
